# MR Fingerprinting—A Radiogenomic Marker for Diffuse Gliomas

**DOI:** 10.3390/cancers14030723

**Published:** 2022-01-30

**Authors:** Elisabeth Springer, Pedro Lima Cardoso, Bernhard Strasser, Wolfgang Bogner, Matthias Preusser, Georg Widhalm, Mathias Nittka, Gregor Koerzdoerfer, Pavol Szomolanyi, Gilbert Hangel, Johannes A. Hainfellner, Wolfgang Marik, Siegfried Trattnig

**Affiliations:** 1 High-Field MR Centre, Department of Biomedical Imaging and Image-Guided Therapy, Medical University of Vienna, 1090 Vienna, Austria; elisabeth.springer@meduniwien.ac.at (E.S.); pedro.delimacardoso@meduniwien.ac.at (P.L.C.); bernhard.strasser@meduniwien.ac.at (B.S.); pavol.szomolanyi@meduniwien.ac.at (P.S.); gilbert.hangel@meduniwien.ac.at (G.H.); siegfried.trattnig@meduniwien.ac.at (S.T.); 2 Institute of Radiology, Hietzing Hospital, 1130 Vienna, Austria; 3 Division of Oncology, Department of Internal Medicine I, Medical University of Vienna, 1090 Vienna, Austria; matthias.preusser@meduniwien.ac.at; 4 Department of Neurosurgery, Medical University of Vienna, 1090 Vienna, Austria; georg.widhalm@meduniwien.ac.at; 5 Siemens Healthineers, 91052 Erlangen, Germany; mathias.nittka@siemens-healthineers.com (M.N.); gregor.koerzdoerfer@siemens-healthineers.com (G.K.); 6 Department of Imaging Methods, Institute of Measurement Science, Slovak Academy of Sciences, 84104 Bratislava, Slovakia; 7 Division of Neuropathology and Neurochemistry, Department of Neurology, Medical University of Vienna, 1090 Vienna, Austria; johannes.hainfellner@meduniwien.ac.at; 8 Division of Neuroradiology and Musculoskeletal Radiology, Department of Biomedical Imaging and Image-Guided Therapy, Medical University of Vienna, 1090 Vienna, Austria; wolfgang.marik@meduniwien.ac.at; 9 Christian Doppler Laboratory for Clinical Molecular MR Imaging, Medical University of Vienna, 1090 Vienna, Austria

**Keywords:** MR fingerprinting, quantitative maps, T1 and T2 relaxation times, gliomas, brain/central nervous system cancers

## Abstract

**Simple Summary:**

MR imaging of brain tumors is still mainly based on contrast images and 2 quantitative parameters (relative Cerebral Blood Volume and Apparent Diffusion Coefficient). It is time consuming and suffers from inter-scanner variability. MR Fingerprinting is a new approach that relies on the principle that each tissue examined evolves its own unique signal fingerprint within one single short sequence. It provides quantitative information about the tissue and allows the generation of contrast images. The purpose of this study was to evaluate the use of MR Fingerprinting as a radiogenomic marker to differentiate gliomas according to the genotypic marker isocitrate dehydrogenase (IDH) mutation. Based on the results of this study MR Fingerprinting seems to have the potential to differentiate IDH-mutant from IDH-wildtype gliomas, which provides a prognostic factor noninvasively. In general MR Fingerprinting leads to a new area of Neuro MRI with the possibility of acquiring quantitative MRI data in a clinically feasible way using only one single short sequence, which may promote molecular precision imaging.

**Abstract:**

(1) Background: Advanced MR imaging (MRI) of brain tumors is mainly based on qualitative contrast images. MR Fingerprinting (MRF) offers a novel approach. The purpose of this study was to use MRF-derived T1 and T2 relaxation maps to differentiate diffuse gliomas according to isocitrate dehydrogenase (IDH) mutation. (2) Methods: Twenty-four patients with histologically verified diffuse gliomas (14 IDH-mutant, four 1p/19q-codeleted, 10 IDH-wildtype) were enrolled. MRF T1 and T2 relaxation times were compared to apparent diffusion coefficient (ADC), relative cerebral blood volume (rCBV) within solid tumor, peritumoral edema, and normal-appearing white matter (NAWM), using contrast-enhanced MRI, diffusion-, perfusion-, and susceptibility-weighted imaging. For perfusion imaging, a T2* weighted perfusion sequence with leakage correction was used. Correlations of MRF T1 and T2 times with two established conventional sequences for T1 and T2 mapping were assessed (a fast double inversion recovery-based MR sequence (‘MP2RAGE’) for T1 quantification and a multi-contrast spin echo-based sequence for T2 quantification). (3) Results: MRF T1 and T2 relaxation times were significantly higher in the IDH-mutant than in IDH-wildtype gliomas within the solid part of the tumor (*p* = 0.024 for MRF T1, *p* = 0.041 for MRF T2). MRF T1 and T2 relaxation times were significantly higher in the IDH-wildtype than in IDH-mutant gliomas within peritumoral edema less than or equal to 1cm adjacent to the tumor (*p* = 0.038 for MRF T1 mean, *p* = 0.010 for MRF T2 mean). In the solid part of the tumor, there was a high correlation between MRF and conventionally measured T1 and T2 values (r = 0.913, *p* < 0.001 for T1, r = 0.775, *p* < 0.001 for T2), as well as between MRF and ADC values (r = 0.813, *p* < 0.001 for T2, r = 0.697, *p* < 0.001 for T1). The correlation was weak between the MRF and rCBV values (r = −0.374, *p* = 0.005 for T2, r = −0.181, *p* = 0.181 for T1). (4) Conclusions: MRF enables fast, single-sequence based, multi-parametric, quantitative tissue characterization of diffuse gliomas and may have the potential to differentiate IDH-mutant from IDH-wildtype gliomas.

## 1. Introduction

The 2016 World Health Organization (WHO) classification of Tumors of the Central Nervous System (CNS) uses genotypic markers in addition to histomorphological characteristics to define different types of diffuse gliomas [1]. Isocitrate dehydrogenase (IDH) mutations are oncogenic and can be found in the lowest-grade gliomas (LGGs), as well as in secondary high-grade gliomas (HGGs) [2,3]. IDH mutation status prediction is essential for individual therapy planning and prognosis of glioma patients [4,5] and may possibly alter therapeutic strategies, as well as the estimation of the urgency of the neurosurgical procedure and of the necessity of an increased extent of resection [6]. Oligodendrogliomas are classified according to their codeletion of chromosomal arms 1p and 19q, which has a beneficial prognostic implication for sensitivity to chemotherapy and overall outcome [3,7]. Methylation of the methylguanine methyltransferase (MGMT) promoter gene is reported to be a useful biomarker for outcomes after chemotherapy [8].

Several MRI methods have been investigated to find imaging features that correlate to these genetic markers, including morphologic properties, such as location and margins of the tumor, calcifications, and contrast enhancement, and the size of the necrotic area of the tumor [3,9,10], as well as functional characteristics assessed through perfusion imaging and diffusion-weighted imaging (DWI) [3,11,12,13]. MR spectroscopy (MRS) has the potential to assess the IDH mutation status [14] and treatment outcome of diffuse gliomas [15,16] by detection of 2-hydroxyglutarate (2-HG) in IDH-mutant gliomas. Moreover, MR amide proton transfer (APT) and diffusion kurtosis imaging (DKI) have been investigated [17,18], as well as arterial spin labeling (ASL) to measure tumor blood flow (TBF) [10]. However, the above-mentioned MR techniques suffer from limitations, which distract from transfer to clinical application [3,19].

Recently, there has been increasing interest in investigating quantitative MRI, such as mapping of T1 and T2 relaxation times [19,20,21,22] and apparent diffusion coefficient (ADC) [23], to develop imaging biomarkers that complement subjective radiological assessment [24]. Conventional quantitative methods for T1 and T2 mapping typically acquire only a single parameter at a time, and their sensitivity to scanner-specific imperfections, such as transmit (B_1_-) field variations, is difficult to control [25].

MR Fingerprinting (MRF), a novel MRI methodology, takes a completely different approach to data acquisition, post-processing, and visualization and consequently may be able to overcome the limitations outlined above [25]. Rather than using a repeated, serial acquisition of data with well-defined contrast for the characterization of individual parameters of interest, a pseudo-randomized and highly-under-sampled signal acquisition is performed, which explicitly avoids signal steady-states within a single, fast sequence. Thus, signals from different tissues are characterized by unique temporal signal evolutions or “fingerprints,” which are, simultaneously, a function of multiple tissue parameters (e.g., T1, T2, and proton density (PD)). Despite the total shorter scan time, the simultaneous acquisition of T1 and T2 is an important feature of MRF, since this is critical for a robust correlation of T1 and T2 values on a pixel level (e.g., by means of a T1-T2 scatterplot); if both parameters are acquired by separate scans over several minutes, a perfect registration of T1 and T2 maps is hardy possible in the presence of subtle patient motion, which is usually unavoidable. Post-acquisition processing involves a pattern-matching algorithm with which to identify the fingerprints that reflect predicted signal evolutions within a pre-calculated dictionary derived from either Bloch simulations or extended phase graphs. The dictionary entry that provides the closest match directly delivers the related tissue parameters of interest, which can be translated into quantitative maps.

The aim of our study was to evaluate MRF as a potential imaging biomarker with which to differentiate the IDH-mutant from the IDH-wildtype in diffuse gliomas and to compare MRF to ADC and leakage-corrected relative cerebral blood volume (rCBV).

## 2. Materials and Methods

This study was performed in compliance with the Declaration of Helsinki and was approved by the local ethics review committee. Written, informed consent was obtained from all study participants prior to examination. Twenty-four patients with histopathologically proven diffuse gliomas (nine female, fifteen male), with a mean age of 58.6 years (age range, 23–77 years), were included between February 2019 and March 2020.

Tumors were pathohistologically classified according to the WHO classification 2016 [24,26]. Fourteen of twenty-four diffuse gliomas showed an IDH-mutation, and ten were the IDH-wildtype. The IDH-mutant group consisted of ten tumors without and four tumors with a 1p/19q-codeletion. Among the IDH-mutant group without a 1p/19q-codeletion, six were diffuse astrocytomas, three anaplastic astrocytomas, and one astrocytoma grade IV. Of the ten IDH-wildtype gliomas, eight were glioblastomas. One glioma in the IDH-wildtype cohort was an anaplastic astrocytoma, which demonstrated a TERT mutation, consistent with a molecular glioblastoma. One tumor in this group was a diffuse astrocytoma. For clinical and histopathological details, see Table 1. One diffuse astrocytoma II, an IDH-mutant, had undergone a previous resection 6 years 4 months before imaging. One anaplastic astrocytoma, an IDH-mutant, had undergone a previous biopsy 5 months before imaging.

The conventional MR brain tumor protocol comprised the sequences and parameters listed in Table 2 and was performed on a 3 T MR scanner (MAGNETOM Trio, Siemens Healthineers, Erlangen, Germany) using a multi-channel head coil with 32 receive channels [19]. For perfusion imaging, a T2* weighted perfusion sequence with leakage correction was used. For leakage correction, a weight-adjusted bolus of 0.2 mL/kg Gadoteridol was administered 3–5 min after an initial weight-adjusted Prebolus of 0.05–01 mL/kg, both with an injection rate of 4 mL/s.

The MRF protocol was performed on a 3 T MR scanner (MAGNETOM PrismaFit, Siemens Healthineers, Erlangen, Germany) with a gradient strength of 80 mT/m and a slew rate of 200 T/m/s, using a head/neck coil with 64 receive channels.

It was not always possible to perform the MRF protocol in our institute on one single day together with the conventional brain tumor protocol because the later one is performed at the neuroradiological division of our department, while MRF imaging is done in a separate examination at the High Field MR Center of our department, which is located separately in another building in the area of the General Hospital of Vienna. In the IDH-wildtype cohort, for the glioblastomas, the MRF protocol and the conventional tumor protocol were performed within a minimum of 0 days, a maximum of three days and a mean of 1.6 days, for one diffuse astrocytoma, IDH-wildtype within 48 days, and for one anaplastic astrocytoma, IDH-wildtype within 21 days. In the IDH-mutant cohort, the two protocols were performed within a minimum of 0 days, a maximum of 38 days, and a mean of 13.8 days.

Intravenous gadolinium-based contrast administration in the conventional protocol was performed either before or a minimum of 12 h after MRF to avoid contamination of MRF datasets [27].

The mean interval between MRI and surgery was 31 days (range 1–118 days).

### 2.1. MR Fingerprinting Protocol

Conventional MRI techniques were performed for localization of the pathologic region and topographic comparison with the T1 and T2 relaxation time maps. In addition to MRF, T1 and T2 maps were also acquired by two more traditional mapping methods for comparison, consisting of a fast double inversion recovery-based MR sequence (‘MP2RAGE’) for T1 quantification and a multi-contrast spin echo-based sequence for T2 quantification [28,29,30]. T1 and T2 fitting procedures were used as they were provided by the scanner integrated post-processing tools (T1: dual flip angle approach; T2: mono-exponential fitting approach). For T2 mapping, the impact of stimulated echoes was reduced by adapted rf-pulse parameters within the sequence (Siemens T2 MapIt sequence), as described in Mendlik et al. [31] Complete MRF protocol is listed in Table 3.

The MRF sequence was based on a 2D Fast Imaging with Steady-state Precession (FISP) acquisition scheme [32,33] with a pre-scan-based B1+ correction [30]. After a non-selective adiabatic inversion pulse, a series of 1500 spiral readouts were acquired at variable TR and flip angles. The spiral acquisition was designed with an under-sampling factor of 48 and a spiral angle increment of 82.5°.

The Siemens AutoAlign scout localizer was used to attain reproducible automated anterior commissure (AC)–posterior commissure (PC) slice alignment.

Voxel-wise T1 and T2 parameters were derived by matching the temporal signal pattern from the acquired 1500 echoes to the entries of a pre-calculated dictionary computed by solving the Bloch equations [25]. T1 and T2 parameter maps were calculated by prototype software integrated into the scanner’s image reconstruction pipeline. The covered parameter range of the dictionary was 10–4500 ms for T1 and 2–3000 ms for T2.

Motion artifacts, particularly those occurring due to through-plane motion, may cause significant deviations of the quantitative parametric values [34,35]. To decrease the likelihood of motion, this study included two consecutive repetitions of the MRF sequence. Furthermore, the MRF prototype used has been modified to include a semi-automatic quality control algorithm to detect the occurrence of motion via automatic calculation of “on-the-fly” difference maps between segments of the signal temporal evolution measured with MRF and their theoretical (expected) evolution. These maps are therefore highly sensitive to head motion. Distinct motion patterns can be visualized to determine whether a slice was motion corrupted or not (with severity levels: “low”, “medium”, and “strong”). All MRF datasets were qualitatively evaluated on a slice-by-slice basis by an observer (G.K.) and labelled accordingly [34]. For region-of-interest (ROI) evaluation, only slices classified with “no” or “low” levels of motion artifacts were used.

### 2.2. Co-Registration

MP2RAGE 3D high resolution morphological images of the conventional brain tumor protocol were used to co-register those of the MRF protocol. The iterative co-registration was performed by the MR Quantitative Tool (MRQT) software prototype (Siemens Healthineers, Erlangen, Germany) via trilinear interpolation.

### 2.3. Region-of-Interest (ROI) Evaluation

**Figure 1:**
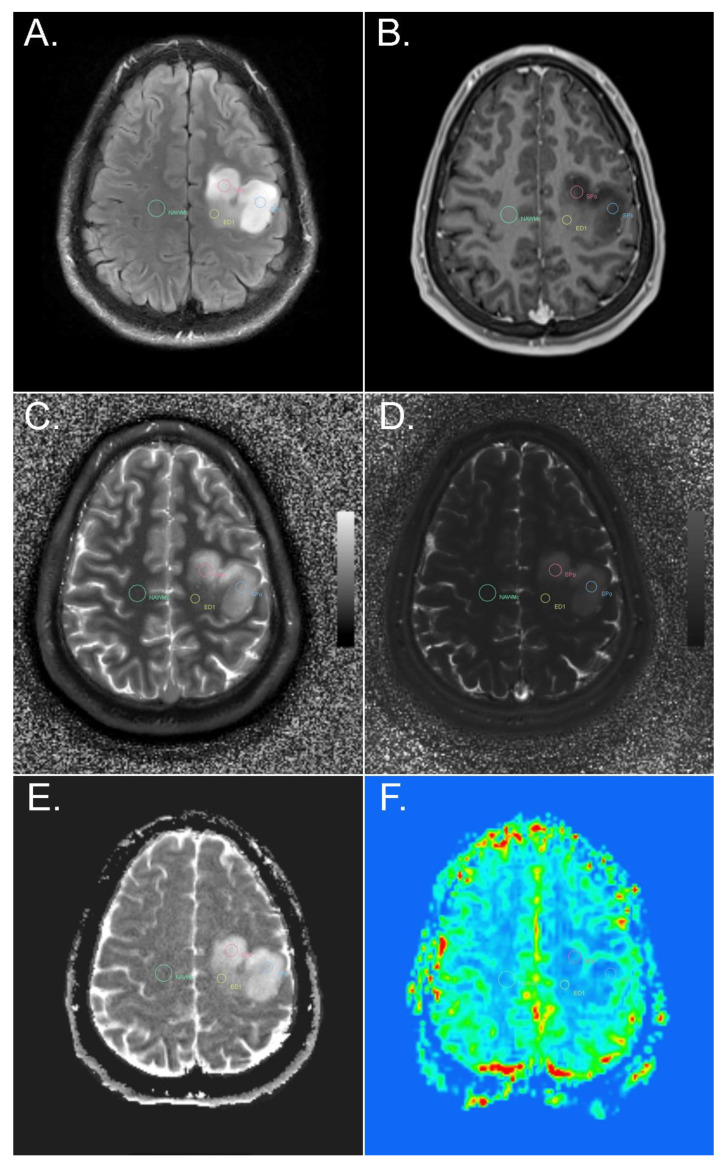
Example of ROI placement in different tumor and peritumoral components of a diffuse astrocytoma, IDH-mutant, in a left precentral location. FLAIR (**A**), T1-weighted MPR post i.v. contrast administration (**B**), MRF T1 (**C**) and T2 (**D**) maps, ADC map (**E**), and perfusion-weighted imaging (**F**). ROIs were placed in the solid tumor part without contrast enhancement and without an increase in rCBV (red and blue circle), in peritumoral edema (yellow circle), and in contralateral NAWM (green circle). SPo, solid part of the tumor without contrast enhancement; ED1, peritumoral edema less than or equal to 1 cm distant from the tumor; NAWMc, contralateral NAWM of the frontal lobe.

**Figure 2:**
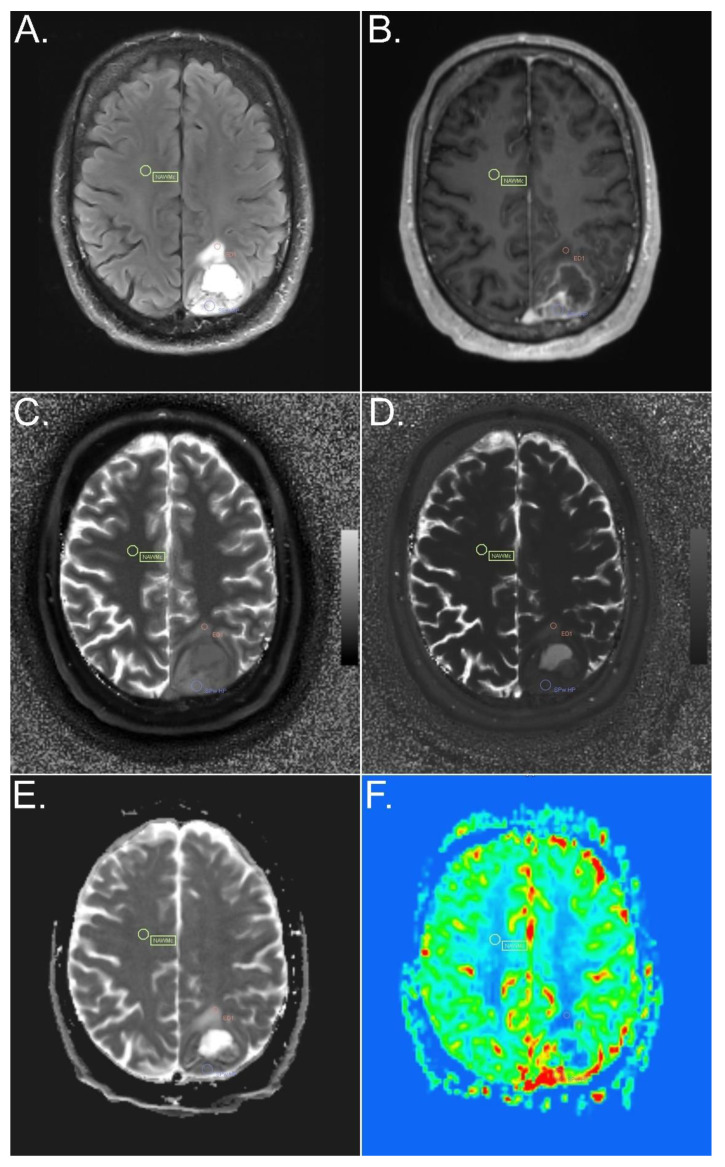
Example of ROI placement in different tumor components of a glioblastoma, IDH-wildtype. FLAIR (**A**), T1-weighted MPR post i.v. contrast administration (**B**), MRF T1 (**C**) and T2 (**D**) maps, ADC map (**E**), and perfusion-weighted imaging (**F**). ROIs were placed in the solid tumor part, in an area with contrast enhancement and an rCBV increase (violet circle), in peritumoral edema (red circle), and in contralateral NAWM (yellow circle). SPw HP, solid part of the tumor with contrast enhancement and with hyperperfusion; ED1, peritumoral edema less than or equal to 1 cm distant from the tumor; NAWMc, contralateral NAWM of the frontal lobe.

The ROIs were selected on multiple slices by two experienced neuroradiologists (E.S. and W.M.) in consensus, based on FLAIR-TSE, T2-TSE, and T1 MP2RAGE of the MRF protocol, and post-contrast T1 MP2RAGE and SWI of the advanced tumor protocol, using the MRQT Prototype: (i) the solid part of the tumor, with and without contrast enhancement, on the rCBV map visually hyperperfused and non-hyperperfused; (ii) peritumoral edema less than or equal to 1 cm distant from the tumor and more than 1 cm distant from the tumor; (iii) perilesional NAWM less than or equal to 1 cm distant from the tumor or from peritumoral edema (if the solid part of the tumor was surrounded by peritumoral edema); and (iv) contralateral NAWM of the frontal lobe (Figure 1 and Figure 2).

Areas of hemorrhage or necrosis were avoided in ROIs drawn in the solid parts of the tumor, edema, and NAWM. ROIs with contrast enhancement and rCBV increase could only be selected in the solid part if present. For the image evaluation, rCBV was judged by the neuroradiologists based on image intensity. In our experience, a standardized ROI placement with the best and easiest possibility to avoid any contamination by gray matter structures, potential microangiopathic changes, or susceptibility effects is best feasible in the frontal lobes due to sufficient space. Age- and gender-related effects on MRF T1 and T2 relaxation times in the brain in asymptomatic volunteers have been well examined by Badve et al. [36].

The number of slices used was dependent on the size and morphology of the tumor, as well as on the localization of the tumor in the brain. As MRF maps are sensitive to motion artifacts, only slices that were, before the neuroradiological assessment, rated “no” or “low” levels of artifacts by an experienced observer could be used. Two to six slices were used for the assessment, with a mean of 3.7 slices.

The number of ROIs, as well, depended on the size and morphology of the tumor. In the vast majority of cases, two or three ROIs were selected in the solid part, if possible, one or two ROIs in different portions within the solid part (contrast-enhancing and on the rCBV map visually hyperperfused, as well as non-contrast-enhancing and non-hyperperfused solid tumor areas). The mean number of ROIs drawn within the solid part was 2.7. Maximally five ROIs were drawn in the solid part, with maximally three ROIs in different portions of the solid part.

Not every tumor exhibited perifocal edema, particularly perifocal edema > 1 cm distant from the solid part (e.g., IDH-mutant, and IDH-mutant, 1p/19q codeleted). Zero to three ROIs were selected in each of the 2 subgroups in perifocal edema (in the vast majority 0–2 ROIs), in both groups of perifocal edema overall 0–5 ROIs (in the vast majority 0–3 ROIs). In addition, in perilesional NAWM, ROI placement was dependent on the morphology of the tumor. One or two ROIs were selected in perilesional NAWM. For perilesional NAWM, to be as exact as possible, if the solid part was surrounded by edema, we created an additional extra group for ROIs less than or equal to 1 cm distant from peritumoral edema.

The ROIs used were circles. The circle size was best adapted to the morphologic conditions of every characteristic region of the tumor by the two experienced neuroradiologists, combining all the available information of the other sequences (quantitative data, as well as contrast images). The mean pixel number over all ROIs was 15.4 pixels, equivalent to 0.3 cm^2^. To compensate for different ROI sizes, a mean value was calculated in every ROI. To guarantee a sufficient data quality of the mean values, a minimal ROI size with a sufficient high number of pixels was chosen.

All selected ROIs were automatically copied to the co-registered images and maps of all other sequences. MRF T1 and T2 relaxation times, ADC values, and leakage-corrected rCBV values were compared. Calculated rCBV values are areas under the R2* curve (rCBF = rCBV/rMTT) [37].The correlations between MRF T1 and T2 values and those from the conventional T1 and T2 mapping sequences, as well as ADC and rCBV values, were assessed in the solid part of the tumor and in peritumoral edema.

### 2.4. Statistical Analysis

All statistical computations were performed using IBM SPSS Statistics for Windows version 26. Metric data are described using the mean and standard error. Multi-level analysis (MLA) was assessed to consider clustered multiple measures per patient when comparing different regions. Bivariate correlation between metric data was calculated using the Pearson correlation coefficient (r). A *p*-value ≤ 0.05 was considered significant. To avoid an error of the second type, no multiplicity corrections were performed.

## 3. Results

### 3.1. IDH-Mutant versus IDH-Wildtype

**Figure 3:**
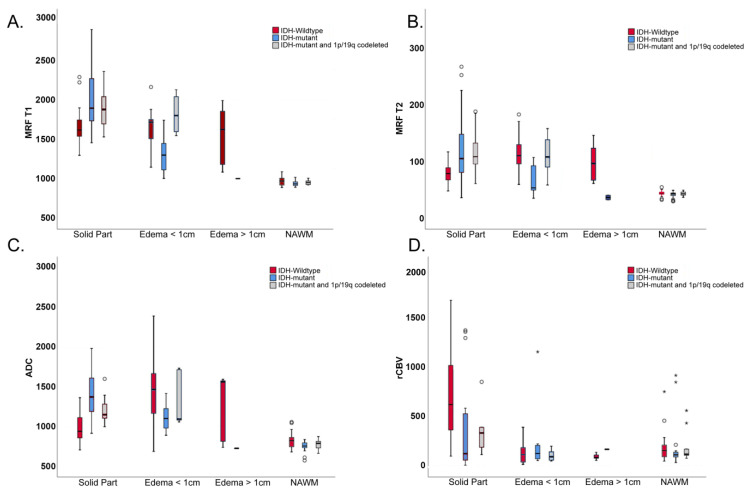
MRF T1 (**A**) and T2 relaxation time values (**B**), ADC values (**C**), and rCBV values (**D**) from different types of tissue (solid part of the tumor, peritumoral edema ≤ 1 cm distant from the tumor and >1 cm distant from the tumor, and NAWM) in IDH-wildtype, IDH-mutant, and IDH-mutant and 1p/19q codeleted diffuse gliomas. Please note the significant difference in MRF T1, MRF T2, and ADC in solid tumor parts between the IDH-mutant and IDH-wildtype. Also note the significant difference in MRF T1 and T2 in peritumoral edema (≤1 cm distant from the solid part) between IDH-mutant and IDH-wildtype.

MRF T1 and T2 values and ADC values of solid tumor parts were significantly higher in the IDH-mutant than in the IDH-wildtype (*p* = 0.024 for MRF T1, *p* = 0.041 for MRF T2, *p* < 0.001 for ADC) (Figure 3A–C). rCBV values in the solid parts of the tumor were higher in the IDH-wildtype than in the IDH-mutant but did not reach statistical significance (*p* = 0.252) (Figure 3D).

In perilesional edema, MRF T1 and T2 values were significantly higher in the IDH-wildtype than in the IDH-mutant (*p* = 0.038 for MRF T1, *p* = 0.010 for MRF T2) (Figure 3A,B). ADC values were higher in the IDH-wildtype than in the IDH-mutant but did not reach statistical significance (*p* = 0.409) (Figure 3C).

For the multinominal logistic regression analysis, within the solid part of the tumor, the IDH-wildtype was correctly assigned in 85.7% by MRF T1, in 85.7% by MRF T2, in 84% by ADC, and in 78.6% by rCBV. IDH-mutant was correctly allocated in 72.7% by MRF T1, in 63.6% by MRF T2, in 77.3% by ADC, and in 70% by rCBV. IDH-mutant with 1p/19q codeletion was not correctly assigned by either of the four variables.

**Figure 4:**
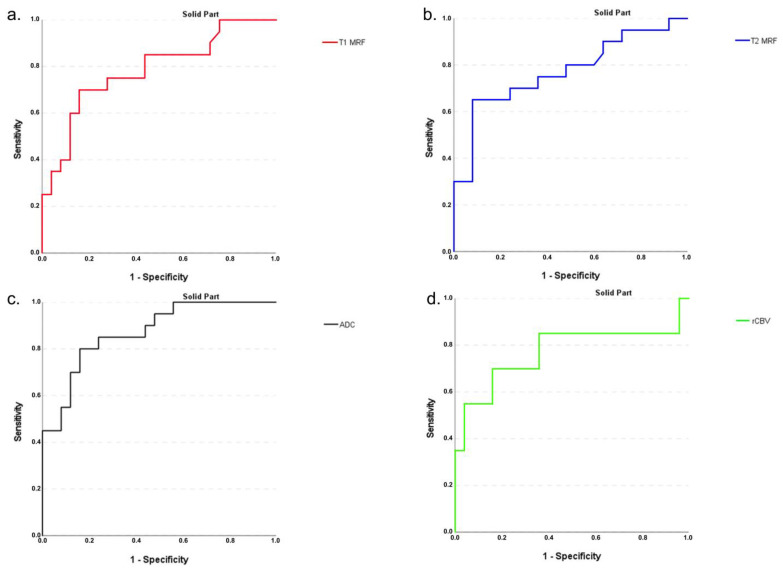
Receiver operating characteristic (ROC) curves to differentiate the solid parts of the IDH mutant (without 1p/19q codeletion) from the IDH-wildtype for the mean of MRF T1 (**a**) and T2 relaxation time values (**b**), ADC values (**c**), and rCBV values (**d**). The area under the curve AUC for MRF T1 (0.791) (**a**) and T2 (0.754) (**b**) shows that differentiation can well be performed, as with ADC (0.875) and rCBV (0.239).

For the ROCs to differentiate the solid parts of the IDH-mutant without 1p/19q codeletion from the IDH-wildtype, the area under the curve (AUC) was 0.791 for the mean of MRF T1 values, and 0.754 for the mean of MRF T2 values. The AUC for the mean of rCBV values was 0.239, for the mean of ADC values 0.875 (Figure 4).

The solid part may be assigned as the IDH-mutant without 1p/19q codeletion, compared to the IDH-wildtype, if MRF T1 values are higher or equal to 1670.9 (with a sensitivity of 0.773, and a 1-specificity of 0.321), and if MRF T2 values are higher or equal to 84.55 (with a sensitivity of 0.727, and a 1-specificity of 0.286).

### 3.2. Low Grade Gliomas (LGG) versus High Grade Gliomas (HGG)

Within the solid part of the tumor, MRF T1 and T2 relaxation times, as well as ADC values were significantly higher in LGGs compared to HGGs (*p* = 0.017 for MRF T1, *p* = 0.002 for MRF T2, and *p* = 0.003 for ADC). RCBV values were significantly higher in HGGs compared to LGGs *(p* < 0.001).

### 3.3. IDH Mutational Status within Different Tumor Grades

**Figure 5:**
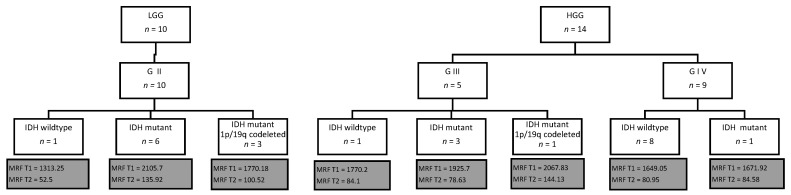
Mean of MRF T1 and T2 relaxation time values in the solid part of the tumor in IDH-wildtype, IDH-mutant, and IDH-mutant and 1p/19q codeleted diffuse gliomas, according to their tumor grade. Within our cohort of ten LGGs, one was an IDH-wildtype and six were IDH-mutants. Within the group of 14 HGGs nine were grade IV, and five were grade III gliomas. The cohort of the nine grade IV gliomas consisted of eight IDH-wildtype and one IDH-mutant gliomas. Due to the low sample size, only a descriptive analysis could be done. Please note lower means of MRF T1 and T2 relaxation time values within the one grade II IDH-wildtype glioma than within the six grade II IDH-mutant gliomas (non-codeleted), and the grade IV IDH-wildtype gliomas. Furthermore, a minimally higher mean of MRF T1 and T2 relaxation time values was observed within the one grade IV IDH-mutant glioma compared to the eight grade IV IDH-wildtype gliomas.

Within our cohort of ten LGGs, one was an IDH-wildtype and six were IDH-mutants. Within the group of 14 HGGs there were nine grade IV gliomas and five grade III gliomas. The cohort of the nine grade IV gliomas consisted of eight IDH-wildtype gliomas and one IDH-mutant, the group of the five grade III gliomas consisted of only one IDH-wildtype glioma and three IDH-mutant gliomas. Due to the low sample size within each group of the mutational status within each tumor grade, only a descriptive analysis could be done (Figure 5).

The mean of MRF T1 and T2 relaxation time values within the only grade II IDH-wildtype glioma (1313.25 ms for MRF T1, 52.5 ms for MRF T2) were lower compared to the six grade II IDH-mutant gliomas (non-codeleted) (2105.7 ms for MRF T1, 135.92 ms for MRF T2) and also lower compared to the eight grade IV IDH-wildtype gliomas (1649.05 ms for MRF T1, 80.95 ms for MRF T2) (Figure 5).

The mean of MRF T1 and T2 relaxation time values within the one grade IV IDH-mutant glioma (1671.92 ms for MRF T1, 84.58 ms for MRF T2) were minimally higher compared to the eight grade IV IDH-wildtype gliomas (1649.05 ms for MRF T1, 80.95 ms for MRF T2).

The mean of MRF T1 relaxation time values within the one grade III IDH-wildtype glioma (1770.2 ms) were lower compared to the three grade III IDH-mutant gliomas (non-codeleted) (1925.7 ms). The mean MRF T2 relaxation time values within the one grade III IDH-wildtype glioma (84.1 ms) were minimally higher compared to the three grade III IDH-mutant gliomas (non-codeleted) (78.63 ms) (Figure 5).

### 3.4. MGMT Methylation Status

There was no significant difference between methylated and unmethylated gliomas within the solid tumor part, either for MRF T1, MRF T2, ADC or for CBV values (*p* = 0.135 for MRF T1, *p* = 0.210 for MRF T2, *p* = 0.330 for ADC, and *p* = 0.661 for rCBV).

### 3.5. Solid Tumor—NAWM

**Figure 6:**
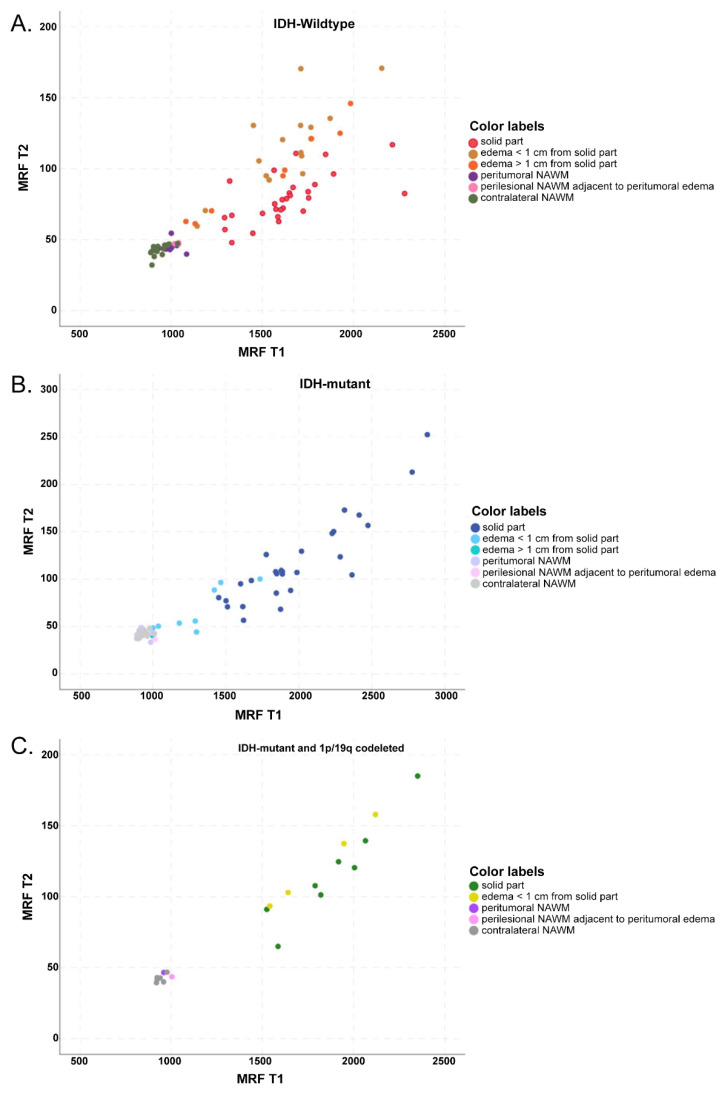
Scatterplot of MRF T1 and T2 relaxation time values in IDH-wildtype (**A**), IDH-mutant (**B**), and IDH-mutant 1p/19q codeleted gliomas (**C**) from different types of tissue (solid part of the tumor; peritumoral edema ≤ 1 cm distant from the tumor and > 1 cm distant from the tumor; perilesional NAWM ≤ 1 cm distant from the tumor or peritumoral edema; and contralateral NAWM). Significant difference for MRF T1 and T2 between solid parts of the tumor and NAWM in the IDH-wildtype and IDH-mutant, including 1p/19q-codeletion. Also notice the significant difference for MRF T1 and T2 between solid tumor components and peritumoral edema in IDH-mutant, and for MRF T2 between solid tumor components and peritumoral edema in the IDH-wildtype (when divided into sub-specified areas of peritumoral edema, significant only in edema ≤ 1 cm distant from the solid part).

MRF T1 and T2 values were significantly higher in the solid parts of the tumor than in NAWM (*p* ≤ 0.001 for MRF T1 and T2 in IDH-wildtype and IDH-mutant, including 1p/19q-codeletion) (Figure 3A,B and Figure 6A–C).

The rCBV values in the IDH-wildtype (*p* < 0.001) were significantly higher in the solid parts of the tumor than in NAWM but not in the IDH-mutant (Figure 3D). For ADC values, the difference was significant only for IDH-mutant, including 1p/19q-codeleted tumors (*p* = 0.003), but not the IDH-wildtype (Figure 3C).

### 3.6. Solid Tumor—Peritumoral Edema

In the IDH-wildtype, MRF T2 and ADC values were generally higher in peritumoral edema than in solid tumor components (*p* = 0.003 for MRF T2, *p* < 0.001 for ADC) (Figure 3B,C), where, when divided into peritumoral edema less than or equal to 1 cm distant from the tumor and peritumoral edema more than 1 cm distant from the tumor, the difference was significant only in edema less than or equal to 1 cm distant from the tumor (*p* = 0.011 for MRF T2, *p* ≤ 0.001 for ADC).

In the IDH-mutant, MRF T1, MRF T2, and ADC values were higher in the solid part than in peritumoral edema, in general (*p* < 0.001 for all three parameters) (Figure 3A–C and Figure 6B).

The rCBV values were significantly higher in solid tumor components than in peritumoral edema in the IDH-wildtype (*p* < 0.001) as well as in IDH-mutant, 1p/19q-codeleted tumors (*p* = 0.007) (Figure 3D).

### 3.7. Contrast-Enhancing and Hyperperfused versus Non-Contrast-Enhancing and Non-Hyperperfused Solid Tumor in IDH-Wildtype Gliomas

We found no statistically significant difference in MRF T1 and T2 values between contrast-enhancing and, on the rCBV map, visually hyperperfused solid tumor areas (Figure 2) and non-contrast-enhancing, visually non-hyperperfused solid tumor areas (Figure 1) (*p* = 0.18 for MRF T1, *p* = 0.134 for MRF T2). ADC values were significantly lower in contrast-enhancing, hyperperfused solid tumor areas than in non-contrast-enhancing, non-hyperperfused solid tumor areas (*p* = 0.014). The rCBV values were significantly higher (*p* < 0.001) in contrast-enhancing, hyperperfused solid tumor areas than in non-contrast-enhancing, non-hyperperfused solid tumor areas.

Only three ROIs could be assessed in the solid tumor part of the tumor with contrast enhancement and without an increase in rCBV (two ROIs in two of the Glioblastomas, IDH-wildtype, and one ROI in the Glioblastoma, IDH mutant). Due to the small sample size, a statistical analysis could not be performed.

### 3.8. Correlation between MRF and Other Advanced MR Methods

In the solid part of the tumor, there was a high correlation between MRF T2 relaxation times and ADC values, with an r = 0.813 (*p* < 0.001), and between MRF T1 relaxation times and ADC values, with an r = 0.697 (*p* < 0.001). The correlation between rCBV values and MRF T2 (r = −0.374, *p* = 0.005) and MRF T1 values was weak and not significant for MRF T1 (r = −0.181, *p* = 0.181). In peritumoral edema, we found similar correlations (Table 4).

### 3.9. Comparison between MRF and Conventional T1 and T2 Mapping

In the solid part of the tumor, there was a high correlation between MRF T1 and conventionally measured T1 values, with an r = 0.913 (*p* < 0.001). There was also a high correlation between MRF T2 and conventionally measured T2 values, with an r = 0.775 (*p* < 0.001).

In peritumoral edema, we found similar correlations (Table 4).

## 4. Discussion

In our study, there were significant differences in MRF T1 and T2 values between IDH-mutant and IDH-wildtype gliomas in the solid tumor part. The only report thus far to compare MRF T1 and T2 values with the mutational status of gliomas was reported by Haubold et al., who investigated MRF in combination with multiparametric PET-MRI [38]. In their cohort of 30 diffuse gliomas, five 1p/19q codeleted tumors were included. They reported a high sensitivity and specificity to discriminate gliomas according to their IDH-mutation status when combining contrast-enhanced T1 images, FLAIR (SPACE) images, and the water-content-based M0 map (MRF M0), but not with T1 and T2 relaxation time values [34].

Then, we found significantly higher MRF T1 and T2 values, as well as ADC values, in LGGs compared to HGGs and accordingly significantly lower rCBV values. Badve et al. [39], using MRF-based T1 and T2 quantification in the solid part of the tumor, did not find a significant difference between glioblastomas and LGGs; however, their cohort of six LGGs consisted of five oligodendrogliomas [3,40] and one oligoastrocytoma, and they compared gliomas based only on their grading but not on their mutational status. Using the same cohort of patients, Dastmalchian et al. found an optimal separation for three tumor groups (the two described groups of gliomas as well as metastases) utilizing texture analysis methods of MRF-derived T1 and T2 maps in peritumoral white matter only and only with two features [41]. In both articles, motion correction was not applied for MRF.

Earlier and improved tumor classification by radiogenomic-based prediction of the mutational status may accelerate prediction of the individual patient prognosis and estimation of the urgency of the neurosurgical procedure, as well as of the necessity of an increased extent of resection. For example, in the case of preoperative suspicion of an LGG IDH wildtype, rapid neurosurgical resection/biopsy with subsequent adjuvant therapy should be planned without delay. Patel et al. found that IDH mutant gliomas benefit most from an extended resection (in contrast to IDH wildtype gliomas) [6]. Their cohort consisted of WHO grade II and III gliomas (IDH mutant, IDH mutant and 1p/19q codeleted, IDH wildtype). If it is preoperatively clear that a glioma is an IDH mutant, a maximal possible safe resection should be performed. In our (due to the small sample sizes in the resulting subgroups) solely descriptive performed analysis, we found lower MRF T1 and T2 relaxation time values within one grade II IDH-wildtype glioma than within the grade II IDH-mutant gliomas (non-codeleted). Furthermore, we found minimally higher MRF T1 and T2 relaxation time values within one grade IV IDH-mutant glioma than within the grade IV IDH-wildtype gliomas.

In our study, the ADC values of solid tumor parts were significantly lower in the IDH-wildtype than in the IDH-mutant glioma. This is in accordance with Hong et al., who also observed higher ADC values in glioblastoma IDH-mutants compared to the IDH-wildtype [42]. This is furthermore in agreement with Cindil et al., who also, within a cohort of HGGs (glioblastomas and anaplastic astrocytomas), observed lower ADC values in the IDH-wildtype than in the IDH-mutant [13].

In our study, ADC values were also significantly lower in contrast-enhancing, hyperperfused than in non-contrast-enhancing, non-hyperperfused solid tumor areas in IDH-wildtype gliomas, which can be explained by either higher cellularity or cytotoxic edema.

In our study, MRF T1 and T2 values in peritumoral edema were significantly higher in IDH-wildtype than in IDH-mutant gliomas. This may have been caused by a higher degree of water content underlying infiltration into the adjacent peritumoral edema in wild-type gliomas and may indicate a larger tumor extension than seen on contrast images.

Furthermore, in the IDH-wildtype, MRF T2 relaxation times and ADC values showed significant differences between solid tumor tissue and peritumoral edema. These findings are in accordance with a study by Hoehn-Berlage et al. In different types of tumors (glioma, schwannoma, neuroblastoma), concordant with our results, they found conventional T2 mapping seemed to be the better parameter to discriminate between a tumor and edema [43]. These results are also consistent with a study by Oh et al. [44], who found significantly higher ADC values and conventionally measured T2 values in edema than in solid tumor parts in HGG patients (grade IV and grade III), as well as in patients with meningiomas or metastases.

We found a strong correlation between MRF T2 relaxation times and ADC values in the solid tumor parts and in peritumoral edema in our study. This may indicate that there is high cellularity and, at the same time, little edema in solid parts of the IDH-wildtype compared to the IDH-mutant, where there may be less cellular density and a higher fluid component (Figure 3B,C). Our findings are in agreement with Oh et al., who also observed a strong correlation between ADC and conventionally measured T2 values in solid tumor parts and edema for gliomas, as well as for metastases or meningiomas [44]. In our cohort of patients suffering from diffuse gliomas, MRF T1 and T2 values showed a good correlation with the conventional T1 and T2 mapping techniques. These results are in good agreement with Jiang et al. [32].

Preliminary results show that MRF used in our study, compared to other, new encoding schemes for accelerated quantitative MRI, acquired quantitative results with increased accuracy in a shorter time compared to established quantitative MRI measurements, without the high vulnerability to measurement errors and system imperfections found in many other fast quantitative methods [25,28,30,32,45]. In addition, MRF has demonstrated a high repeatability for relaxation time assessment over a period of more than a month [45]. In vivo MRF T1 and T2 relaxation times in solid brain compartments acquired on different scanners at 3 T were found to vary less than 8% [45]. Since MR fingerprinting provides quantitative relaxation parameters of the brain tissue, it is inherently more sustainable in terms of inter-scan variability than conventional contrast-based MR imaging. For example, both the absolute signal intensity and tissue contrast of conventional imaging are affected by the receive coil sensitivity profiles and the transmit field (B_1_-field) variations, which are particularly prominent at 3T field strength inside the human head.

In quantitative MRI methods, such as MRF, motion artifacts may be very subtle to detect, but largely influence the estimation of the parametric maps (e.g., particularly in T2). In our study, motion detection was performed in a qualitative assessment, as described by Körzdörfer et al. [34] which limited the deviation in T2 values due to motion to below 10%—a value far below the changes expected in the presence of pathological tissue [39,45]. The remaining challenge to make MRF routinely applicable will be the manner of this necessary assessment, respectively exclusion of motion artifacts in the acquired MRF slices. This was done by an independent experienced observer in our study. Speaking of a multiplicity of images of examined patients, a potential solution contemplated to overcome this challenge could possibly be a machine learning-based automatic detection of motion artifacts in the slices.

### Limitations of the Study

A limitation of this study is the small patient sample, and further larger clinical trials will be needed for confirmation of our results.

The clinical, advanced brain tumor protocol and the MRF protocol were performed on two different models of a 3 T MR scanner from a single manufacturer using two different head coils, but it has been shown previously in a multi-center study that MRF results are very stable across different 3 T MR hardware setups of the same manufacturer [46].

Scanning of the tumor protocol and the MRF sequence could not always be performed on the same day. In future studies, MRF should be integrated into the clinical brain tumor protocol.

Due to tumor heterogeneity, the ROIs may not have covered the whole spectrum of tumor characteristics.

A slice thickness of 5 mm was chosen to keep it identical with previous published studies investigating the quantitative performance of MRF (in particular, precision). Thinner slices are possible from a technical standpoint; however, initial experiments showed a decrease in precision (increased ‘noise’ in the T1 and T2 maps), i.e., the impact of thinner slices on the MRF results could not be answered within the scope of our study. Future implementations of MRF will seek to address current restrictions, with improved in-plane resolution and 3D acquisition capabilities, while improving processing speeds and patient comfort [47,48,49,50,51].

## 5. Conclusions

We conclude that MRF allows fast multi-parametric, quantitative tissue characterization of gliomas. It may be of additional benefit for the discrimination of the IDH mutation status of diffuse gliomas, with an emphasis on prognosis and individual therapy planning for glioma patients, also including influence on the decision of the urgency of the neurosurgical procedure. This may offer a promising new approach toward imaging biomarkers in neuroimaging.

## Figures and Tables

**Table 1:** Patient Characteristics.

Neuropathological Tumor Type (WHO 2016)	Neuropathological Tumor Grade (WHO 2016)	MGMPR Promoter Methylation Status	Age	Gender
Diffuse astrocytoma, IDH-mutant	WHO grade II	Methylated	23	M
Diffuse astrocytoma, IDH-mutant	WHO grade II	Methylated	33	M
Diffuse astrocytoma, IDH-mutant	WHO grade II	Unmethylated	54	F
Diffuse astrocytoma, IDH-mutant	WHO grade II	Methylated	77	F
Diffuse astrocytoma, IDH-mutant	WHO grade II	Methylated	46	F
Diffuse astrocytoma, IDH-mutant	WHO grade II	Unmethylated	57	M
Diffuse astrocytoma, IDH-wildtype	WHO grade II	Unmethylated	27	M
Anaplastic astrocytoma, IDH-mutant	WHO grade III	Unmethylated	59	M
Anaplastic astrocytoma, IDH-mutant	WHO grade III	Methylated	29	M
Anaplastic astrocytoma, IDH-mutant	WHO grade III	Methylated	28	F
Anaplastic astrocytoma, IDH-wildtype	WHO grade III	Unmethylated	65	F
Glioblastoma, IDH-mutant	WHO grade IV	Methylated	45	F
Glioblastoma, IDH-wildtype	WHO grade IV	Unmethylated	47	F
Glioblastoma, IDH-wildtype	WHO grade IV	Unmethylated	58	M
Glioblastoma, IDH-wildtype	WHO grade IV	Unmethylated	59	F
Glioblastoma, IDH-wildtype	WHO grade IV	Methylated	52	M
Glioblastoma, IDH-wildtype	WHO grade IV	Unmethylated	59	M
Glioblastoma, IDH-wildtype	WHO grade IV	Methylated	71	M
Glioblastoma, IDH-wildtype	WHO grade IV	Methylated	61	M
Glioblastoma, IDH-wildtype	WHO grade IV	Methylated	62	M
Oligoendroglioma, IDH-mutant and 1p/19q-codeleted	WHO grade II	Methylated	52	M
Oligoendroglioma, IDH-mutant and 1p/19q-codeleted	WHO grade II	Methylated	38	F
Oligoendroglioma, IDH-mutant and 1p/19q-codeleted	WHO grade II	Methylated	61	M
Anaplastic oligodendroglioma, IDH-mutant and 1p/19q-codeleted	WHO grade III	Methylated	51	M

**Table 2:** Conventional MR brain tumor protocol used for 3 T.

Examination Parameters	2D ax T2 FLAIR	2D T2 ax	DWI ax	3D SWI ax	3D T1 ax pre	2D T2 cor	PWI ax	3D T1 ax Post	3D FLAIR
	TSE + IR	TSE	EPI-SE	GRE	MPRAGE	TSE	SS-EPI	MPRAGE	TSE + IR
Voxel dimensions	0.9 × 0.9	0.8 × 0.6	1.8 × 1.8	0.9 × 0.9	1 × 1	0.4 × 0.4	1.8 × 1.8	1 × 1	1 × 1
Matrix size	256 × 256	250 × 384	128 × 128	256 × 192	256 × 256	531 × 640	128 × 128	256 × 256	256 × 256
No. slices	36	40	30	80	192	56	19	192	176
Field of view [mm^2^]	230	210	230	230	220	230	230	220	250
Slice thickness, mm	4	3	5	1.75	1	3	5	1	0.9
TE [ms]	100	88	78	20	3.79	115	32	3.79	393
TI [ms]	2500	-	-	-	1100	-	-	-	2050
TR [ms]	9220	3490	4000	28	1800	4290	1400	1800	7000
TA, [min:s]	4:38	1:25	1:38	3:52	5:44	3:40	1:17	5:44	3:39
GRAPPA factor	-	-	2	2	-	2	2	-	-
BW/pixel, [Hz/pixel]	170	199	1502	120	200	176	1346	200	651
FA [°]	150	120	-	15	12	120	90	12	T2var
Fat saturation	yes	No	Yes	No	No	No	Yes	No	Yes

2D T2 FLAIR TSE, 2D T2 weighted fluid-attenuated inversion recovery turbo spin echo; 2D T2 TSE, 2D T2 weighted turbo spin echo; DWI, diffusion weighted echo-planar spin echo imaging; 3D SWI, 3D susceptibility weighted gradient recalled echo imaging; 3D T1 MPRAGE (pre-/post), 3D magnetization prepared rapid acquisition gradient echo sequence pre- and post-contrast injection, post-contrast images are collected with equivalent parameters to pre-contrast images; PWI, perfusion weighted imaging with single-shot echo planar imaging.

**Table 3:** MRF protocols used for 3T.

Examination Parameters	2D ax T2 FLAIR	2D T2 ax	3D T1 Sag	2D Multi-Echo Spin Echo	MRF
	TSE + IR	TSE	MP2RAGE (T1 map)	(T2 map)	
Voxel dimensions [mm^2^]	0.6 × 0.6	0.7 × 0.7	1.0 × 1.0	0.7 × 0.7	1.0 × 1.0
Matrix size	384 × 276	320 × 240	256 × 216	320 × 257	256 × 256
No. slices	10	23	160	10	10–13
Field of view [mm^2^]	230 × 166	230 × 170	256 × 216	230 × 180	256 × 256
Slice thickness [mm]	5.0	5.0	1.0	5.0	5.0
TE [ms]	126	118	2.98	12.6, 25.2, … 201.6	2.0
TI [ms]	2500	–	700, 2500	–	21.0
TR [ms]	8500	4890	5000	2100	12.14–15.00 (varied by sequence)
TA [min:sec]	3:43	1:25	8:02	3:38	3:51–4:51
Acceleration factor	1 (turbo factor: 19)	2	2	3	24 (inner k-space), 48 (outer k-space)
BW/pixel [Hz/pixel]	140	130	240	150	RX-Bandwidth: 400 kHz
FA [°]	180	180	4, 5	180	0–74 (varied by sequence)
Fat saturation	Yes	–	No	No	no

FLAIR fluid-attenuated inversion recovery; TSE, turbo spin echo; MP2RAGE, magnetization prepared 2 rapid acquisition gradient echoes; MRF, Magnetic Resonance Fingerprinting.

**Table 4:** Tumor and peritumoral edema.

Tumor and Peritumoral Edema	Quantitative Parameter	MRFT1 Mean	MRFT2 Mean
Solid part	T1 mean	0.913	
		<0.001	
	T2 mean		0.775
			<0.001
	ADC mean	0.697	0.813
		<0.001	<0.001
	rCBV mean	−0.181	−0.374
		0.181	0.005
Edema ≤ 1 cm adjacent to the solid part	T1 mean	0.882	
		<0.001	
	T2 mean		0.884
			<0.001
	ADC mean	0.742	0.900
		<0.001	<0.001
	rCBV mean	−0.174	−0.223
		0.376	0.254
Edema > 1 cm adjacent to the solid part	T1 mean	0.983	
		<0.001	
	T2 mean		0.983
			<0.001
	ADC mean	0.810	0.786
		0.015	0.021
	rCBV mean	<0.001	−0.050
		1.000	0.898

Shows correlations between MR Fingerprinting T1 and T2 values, conventionally acquired T1 and T2 values, ADC and CBV values in the solid part of the tumor, and in peritumoral edema (Correlation coefficient and Significance).

## Data Availability

All data are available on request to the reviewers.

## References

[1] Louis D.N., Perry A., Wesseling P., Brat D.J., Cree I.A., Figarella-Branger D., Hawkins C., Ng H.K., Pfister S.M., Reifenberger G. (2021). The 2021 WHO Classification of Tumors of the Central Nervous System: A summary. Neuro Oncol..

[2] Cohen A.L., Holmen S.L., Colman H. (2013). IDH1 and IDH2 mutations in gliomas. Curr. Neurol. Neurosci. Rep..

[3] Smits M., van den Bent M.J. (2017). Imaging Correlates of Adult Glioma Genotypes. Radiology.

[4] Yan H., Parsons D.W., Jin G., McLendon R., Rasheed B.A., Yuan W., Kos I., Batinic-Haberle I., Jones S., Riggins G.J. (2009). IDH1 and IDH2 mutations in gliomas. N. Engl. J. Med..

[5] Sanson M., Marie Y., Paris S., Idbaih A., Laffaire J., Ducray F., El Hallani S., Boisselier B., Mokhtari K., Hoang-Xuan K. (2009). Isocitrate dehydrogenase 1 codon 132 mutation is an important prognostic biomarker in gliomas. J. Clin. Oncol..

[6] Patel S.H., Bansal A.G., Young E.B., Batchala P.P., Patrie J.T., Lopes M.B., Jain R., Fadul C.E., Schiff D. (2019). Extent of Surgical Resection in Lower-Grade Gliomas: Differential Impact Based on Molecular Subtype. AJNR Am. J. Neuroradiol..

[7] Van den Bent M.J., Brandes A.A., Taphoorn M.J., Kros J.M., Kouwenhoven M.C., Delattre J.Y., Bernsen H.J., Frenay M., Tijssen C.C., Grisold W. (2013). Adjuvant procarbazine, lomustine, and vincristine chemotherapy in newly diagnosed anaplastic oligodendroglioma: Long-term follow-up of EORTC brain tumor group study 26951. J. Clin. Oncol..

[8] Wick W., Platten M., Meisner C., Felsberg J., Tabatabai G., Simon M., Nikkhah G., Papsdorf K., Steinbach J.P., Sabel M. (2012). Temozolomide chemotherapy alone versus radiotherapy alone for malignant astrocytoma in the elderly: The NOA-08 randomised, phase 3 trial. Lancet Oncol..

[9] Ellingson B.M., Lai A., Harris R.J., Selfridge J.M., Yong W.H., Das K., Pope W.B., Nghiemphu P.L., Vinters H.V., Liau L.M. (2013). Probabilistic radiographic atlas of glioblastoma phenotypes. AJNR Am. J. Neuroradiol..

[10] Yamashita K., Hiwatashi A., Togao O., Kikuchi K., Hatae R., Yoshimoto K., Mizoguchi M., Suzuki S.O., Yoshiura T., Honda H. (2016). MR Imaging-Based Analysis of Glioblastoma Multiforme: Estimation of IDH1 Mutation Status. AJNR Am. J. Neuroradiol..

[11] Kickingereder P., Sahm F., Radbruch A., Wick W., Heiland S., Von Deimling A., Bendszus M., Wiestler B. (2015). IDH mutation status is associated with a distinct hypoxia/angiogenesis transcriptome signature which is non-invasively predictable with rCBV imaging in human glioma. Sci. Rep..

[12] Suh C.H., Kim H.S., Jung S.C., Choi C.G., Kim S.J. (2018). Clinically Relevant Imaging Features for MGMT Promoter Methylation in Multiple Glioblastoma Studies: A Systematic Review and Meta-Analysis. AJNR Am. J. Neuroradiol..

[13] Cindil E., Sendur H.N., Cerit M.N., Erdogan N., Celebi F., Dag N., Celtikci E., Inan A., Oner Y., Tali T. (2021). Prediction of IDH Mutation Status in High-grade Gliomas Using DWI and High T1-weight DSC-MRI. Acad. Radiol..

[14] Choi C., Ganji S.K., DeBerardinis R.J., Hatanpaa K.J., Rakheja D., Kovacs Z., Yang X.L., Mashimo T., Raisanen J.M., Marin-Valencia I. (2012). 2-hydroxyglutarate detection by magnetic resonance spectroscopy in IDH-mutated patients with gliomas. Nat. Med..

[15] Andronesi O.C., Loebel F., Bogner W., Marjańska M., Vander Heiden M.G., Iafrate A.J., Dietrich J., Batchelor T.T., Gerstner E.R., Kaelin W.G. (2016). Treatment Response Assessment in IDH-Mutant Glioma Patients by Noninvasive 3D Functional Spectroscopic Mapping of 2-Hydroxyglutarate. Clin. Cancer Res..

[16] Andronesi O.C., Arrillaga-Romany I.C., Ly K.I., Bogner W., Ratai E.M., Reitz K., Iafrate A.J., Dietrich J., Gerstner E.R., Chi A.S. (2018). Pharmacodynamics of mutant-IDH1 inhibitors in glioma patients probed by in vivo 3D MRS imaging of 2-hydroxyglutarate. Nat. Commun..

[17] Xu Z., Ke C., Liu J., Xu S., Han L., Yang Y., Qian L., Liu X., Zheng H., Lv X. (2021). Diagnostic performance between MR amide proton transfer (APT) and diffusion kurtosis imaging (DKI) in glioma grading and IDH mutation status prediction at 3T. Eur. J. Radiol..

[18] Jiang S., Zou T., Eberhart C.G., Villalobos M.A., Heo H.Y., Zhang Y., Wang Y., Wang X., Yu H., Du Y. (2017). Predicting IDH mutation status in grade II gliomas using amide proton transfer-weighted (APTw) MRI. Magn. Reason. Med..

[19] Thust S.C., Heiland S., Falini A., Jäger H.R., Waldman A.D., Sundgren P.C., Godi C., Katsaros V.K., Ramos A., Bargallo N. (2018). Glioma imaging in Europe: A survey of 220 centres and recommendations for best clinical practice. Eur. Radiol..

[20] Nakai K., Nawashiro H., Shima K., Kaji T., Katayama Y., Maeda T., Kuroiwa T. (2013). An Analysis of T2 Mapping on Brain Tumors. Brain Edema XV. Acta Neurochirurgica Supplement.

[21] Hattingen E., Jurcoane A., Daneshvar K., Pilatus U., Mittelbronn M., Steinbach J.P., Bähr O. (2013). Quantitative T2 mapping of recurrent glioblastoma under bevacizumab improves monitoring for non-enhancing tumor progression and predicts overall survival. Neuro Oncol..

[22] Lescher S., Jurcoane A., Veit A., Bähr O., Deichmann R., Hattingen E. (2015). Quantitative T1 and T2 mapping in recurrent glioblastomas under bevacizumab: Earlier detection of tumor progression compared to conventional MRI. Neuroradiology.

[23] Min Z.G., Niu C., Rana N., Ji H.M., Zhang M. (2013). Differentiation of pure vasogenic edema and tumor-infiltrated edema in patients with peritumoral edema by analyzing the relationship of axial and radial diffusivities on 3.0T MRI. Clin. Neurol. Neurosurg..

[24] European Society of Radiology (2013). ESR statement on the stepwise development of imaging biomarkers. Insights Imaging.

[25] Ma D., Gulani V., Seiberlich N., Liu K., Sunshine J.L., Duerk J.L., Griswold M.A. (2013). Magnetic resonance fingerprinting. Nature.

[26] Louis D.N., Perry A., Reifenberger G., Von Deimling A., Figarella-Branger D., Cavenee W.K., Ohgaki H., Wiestler O.D., Kleihues P., Ellison D.W. (2016). The 2016 World Health Organization Classification of Tumors of the Central Nervous System: A summary. Acta Neuropathol..

[27] Saake M., Schmidle A., Kopp M., Hanspach J., Hepp T., Laun F.B., Nagel A.M., Dörfler A., Uder M., Bäuerle T. (2019). MRI Brain Signal Intensity and Relaxation Times in Individuals with Prior Exposure to Gadobutrol. Radiology.

[28] Crawley P.A., Henkelman R.M. (1988). A comparison of one-shot and recovery methods in T1 imaging. Magn. Reason. Med..

[29] Poon C.S., Henkelman R.M. (1992). Practical T2 quantitation for clinical applications. J. Magn. Reason. Imaging.

[30] Marques J.P., Kober T., Krueger G., van der Zwaag W., Van de Moortele P.F., Gruetter R. (2010). MP2RAGE, a self bias-field corrected sequence for improved segmentation and T1-mapping at high field. Neuroimage.

[31] Mendlik T., Faber S.C., Weber J., Hohe J., Rauch E., Reiser M., Glaser C. (2004). T2 quantitation of human articular cartilage in a clinical setting at 1.5 T: Implementation and testing of four multiecho pulse sequence designs for validity. Investig. Radiol..

[32] Jiang Y., Ma D., Seiberlich N., Gulani V., Griswold M.A. (2015). MR fingerprinting using fast imaging with steady state precession (FISP) with spiral readout. Magn. Reason. Med..

[33] Yokota Y., Okada T., Fushimi Y., Yamamoto A., Nakajima S., Fujimoto K., Oshima S., Koerzdoerfer G., Nittka M., Pfeuffer J. (2020). Acceleration of 2D-MR fingerprinting by reducing the number of echoes with increased in-plane resolution: A volunteer study. Magn. Reson. Mater. Phys. Biol. Med..

[34] Körzdörfer G., Cardoso P.L., Bär P., Kitzer S., Bogner W., Trattnig S., Nittka M. Data-driven motion detection for MR Fingerprinting. Proceedings of the ISMRM & SMRT Virtual Conference & Exhibition.

[35] Yu Z., Zhao T., Assländer J., Lattanzi R., Sodickson D.K., Cloos M.A. (2018). Exploring the sensitivity of magnetic resonance fingerprinting to motion. Magn. Reason. Imaging.

[36] Badve C., Yu A., Rogers M., Ma D., Liu Y., Schluchter M., Sunshine J., Griswold M., Gulani V. (2015). Simultaneous T1 and T2 Brain Relaxometry in Asymptomatic Volunteers using Magnetic Resonance Fingerprinting. Tomography.

[37] Paulson E.S., Schmainda K.M. (2008). Comparison of dynamic susceptibility-weighted contrast-enhanced MR methods: Recommendations for measuring relative cerebral blood volume in brain tumors. Radiology.

[38] Haubold J., Demircioglu A., Gratz M., Glas M., Wrede K., Sure U., Antoch G., Keyvani K., Nittka M., Kannengiesser S. (2020). Non-invasive tumor decoding and phenotyping of cerebral gliomas utilizing multiparametric (18)F-FET PET-MRI and MR Fingerprinting. Eur. J. Nucl. Med. Mol. Imaging.

[39] Badve C., Yu A., Dastmalchian S., Rogers M., Ma D., Jiang Y., Margevicius S., Pahwa S., Lu Z., Schluchter M. (2017). MR Fingerprinting of Adult Brain Tumors: Initial Experience. AJNR Am. J. Neuroradiol..

[40] Koeller K.K., Rushing E.J. (2005). Oligodendroglioma and its variants: Radiologic-pathologic correlation. Radiographics.

[41] Dastmalchian S., Kilinc O., Onyewadume L., Tippareddy C., McGivney D., Ma D., Griswold M., Sunshine J., Gulani V., Barnholtz-Sloan J.S. (2021). Radiomic analysis of magnetic resonance fingerprinting in adult brain tumors. Eur. J. Nucl. Med. Mol. Imaging.

[42] Hong E.K., Choi S.H., Shin D.J., Jo S.W., Yoo R.E., Kang K.M., Yun T.J., Kim J.H., Sohn C.H., Park S.H. (2018). Radiogenomics correlation between MR imaging features and major genetic profiles in glioblastoma. Eur. Radiol..

[43] Hoehn-Berlage M., Bockhorst K. (1994). Quantitative magnetic resonance imaging of rat brain tumors: In vivo NMR relaxometry for the discrimination of normal and pathological tissues. Technol. Health Care.

[44] Oh J., Cha S., Aiken A.H., Han E.T., Crane J.C., Stainsby J.A., Wright G.A., Dillon W.P., Nelson S.J. (2005). Quantitative apparent diffusion coefficients and T2 relaxation times in characterizing contrast enhancing brain tumors and regions of peritumoral edema. J. Magn. Reason. Imaging.

[45] Jiang Y., Ma D., Keenan K.E., Stupic K.F., Gulani V., Griswold M.A. (2017). Repeatability of magnetic resonance fingerprinting T1 and T2 estimates assessed using the ISMRM/NIST MRI system phantom. Magn. Reason. Med..

[46] Körzdörfer G., Kirsch R., Liu K., Pfeuffer J., Hensel B., Jiang Y., Ma D., Gratz M., Bär P., Bogner W. (2019). Reproducibility and Repeatability of MR Fingerprinting Relaxometry in the Human Brain. Radiology.

[47] Ma D., Pierre E.Y., Jiang Y., Schluchter M.D., Setsompop K., Gulani V., Griswold M.A. (2016). Music-based magnetic resonance fingerprinting to improve patient comfort during MRI examinations. Magn. Reson. Med..

[48] Pierre E.Y., Ma D., Chen Y., Badve C., Griswold M.A. (2016). Multiscale reconstruction for MR fingerprinting. Magn. Reson. Med..

[49] Assländer J., Cloos M.A., Knoll F., Sodickson D.K., Hennig J., Lattanzi R. (2018). Low rank alternating direction method of multipliers reconstruction for MR fingerprinting. Magn. Reason. Med..

[50] Ma D., Pierre E.Y., McGivney D., Mehta B., Chen Y., Jiang Y., Griswold M. Applications of low rank modeling to fast 3D magnetic resonance fingerprinting (MRF). Proceedings of the ISMRM, 25th Annual Meeting and Exhibition.

[51] Kiselev V.G., Korzdorfer G., Gall P. (2021). Toward Quantification: Microstructure and Magnetic Resonance Fingerprinting. Investig. Radiol..

